# Correction: Nicotine promotes brain metastasis by polarizing microglia and suppressing innate immune function

**DOI:** 10.1084/jem.2019113101262026c

**Published:** 2026-02-05

**Authors:** Shih-Ying Wu, Fei Xing, Sambad Sharma, Kerui Wu, Abhishek Tyagi, Yin Liu, Dan Zhao, Ravindra Pramod Deshpande, Yusuke Shiozawa, Tamjeed Ahmed, Wei Zhang, Michael Chan, Jimmy Ruiz, Thomas W. Lycan, Andrew Dothard, Kounosuke Watabe

Vol. 217, No. 8 | https://doi.org/10.1084/jem.20191131 | June 4, 2020

The authors regret that, in the originally published version of their article, the left and middle "Nico+PTL" representative mouse images in Fig. 6 I and the right "Nicotine" representative mouse image in Fig. S2 K were inadvertently duplicated from Fig. 2 E and Fig. S2 B, respectively. These errors do not affect the findings of the manuscript, as the correct data were used for all quantitative analyses and are accurately presented in the graphs, and the figure legends remain unchanged. The original and corrected Fig. 6 and Fig. S2 are shown here. The HTML and PDF versions of this paper have been corrected. The errors remain only in print and in PDFs downloaded before January 28, 2026.

**Figure:**
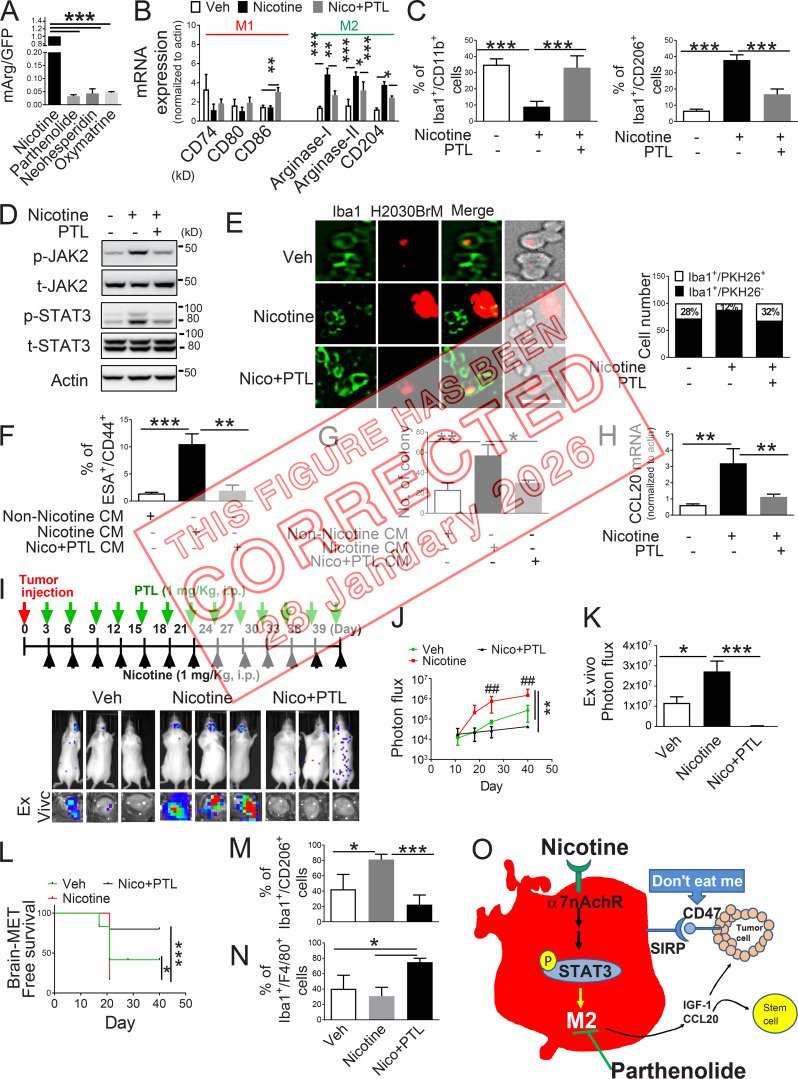


**Figure 6.**
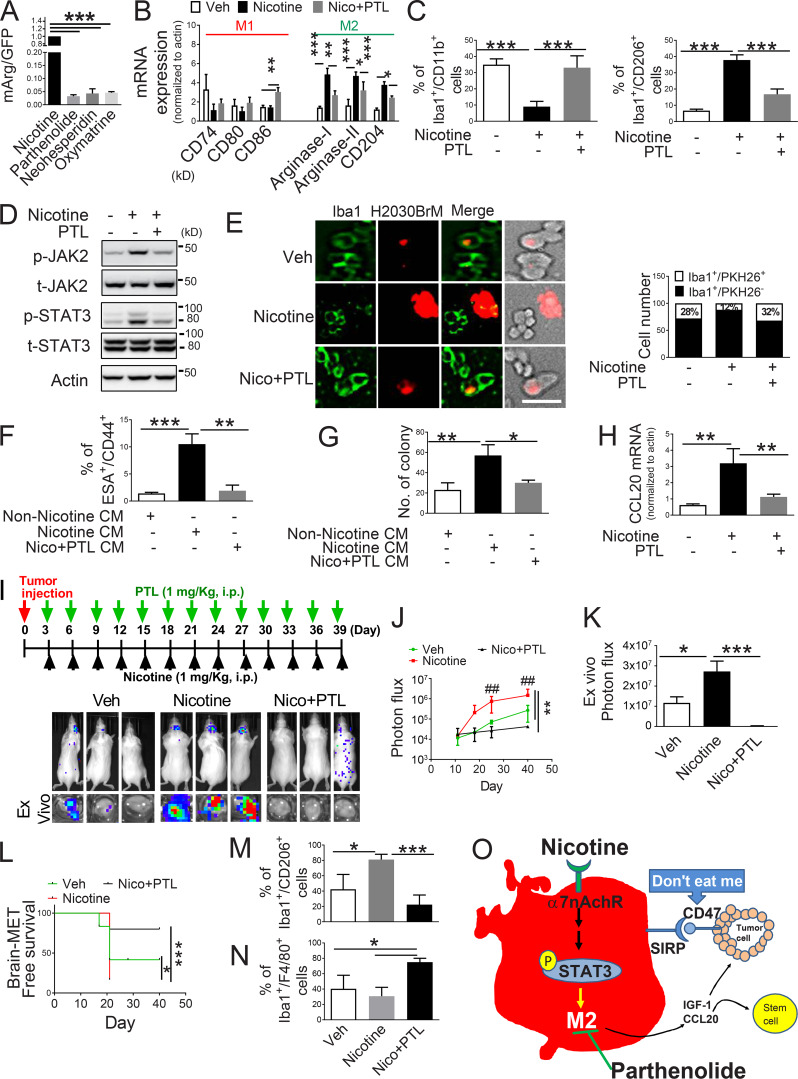
**PTL suppresses brain tumor progression by blocking nicotine-induced M2 microglia polarization. (A)** Human microglia cells (HMC3) with the Arg1 reporter plasmid were cultured in the presence or absence of compounds that were identified as the top three most effective inhibitors for Arg1 during our initial screening (see Materials and methods). After 48 h of incubation, luciferase reporter activity was measured (*n* = 4/group). **(B)** Expression of surface markers of M1/M2 microglia was examined by qRT-PCR after microglial cells were treated with or without nicotine plus PTL (*n* = 4/group). **(C)** The same set of samples in B was evaluated for quantification of Iba1^+^/CD11b^+^ (M1) cells and Iba1^+^/CD206^+^ (M2) cells by FACS (*n* = 4/group). **(D)** The same set of samples in C was examined for quantification of the protein expression of JAK2 and STAT3 by Western blot. **(E)** Human microglial (HMC3) cells (green) with or without nicotine treatment (1 µM) in the presence or absence of PTL (1 µM) were incubated with PKH26-labeled H2030BrM cells (red) for 24 h and photographed (left panels), followed by measurement of the microglial phagocytic activity (right panel; *n* = 4/group). Scale bar, 10 µm. **(F)** CM was prepared from human microglia (HMC3) treated with or without nicotine and PTL. The CM was added to the culture of H2030BrM, and cells were incubated for 48 h followed by evaluation of CSC population by FACS. Non-nicotine, nicotine, or Nico+PTL CM: microglia were treated with PBS, nicotine, or nicotine plus PTL for 24 h. They were then washed twice with PBS and incubated in the fresh DMEM/F12 medium supplemented with 2% FBS for a further 24 h (*n* = 4/group). **(G)** For the same set of samples as F, colony-forming ability was also measured (*n* = 4/group). **(H)** Human microglia were treated with or without nicotine (1 µM) in the presence or absence of PTL for 24 h, followed by assessment of the expression of CCL20 by qRT-PCR (*n* = 4/group). **(I)** The mouse lung cancer cells, LL/2, were intracardially injected into wild-type BALB/c mice. After 3 d of intracranial transplantation of LL/2 cells, mice received nicotine (1 mg/kg) plus PTL (1 mg/kg) by an intraperitoneal injection every 3 d for 40 d. Upper panel: BLI images of representative mice from each experimental group at day 40. Lower panel: total photon flux of ex vivo brain metastatic lesions was measured by BLI at the endpoint (day 40; *n* = 9/group). **(J)** Quantitative data of BLI in the brain regions are shown (*n* = 9/group). **(K)** Ex vivo signals in the whole brains at the end point were quantified. **(L)** The Kaplan–Meier analysis of brain metastasis–free survival was performed (*n* = 9/group). **(M and N)** Metastatic brain tumors in I were isolated from the brain and were examined by FACS for M2 (M) and M1 (N) microglial polarization (*n* = 9/group). **(O)** A proposed model illustrating a nicotine-induced brain metastasis (*n* = 9/group). The data are presented as the mean ± SD. *, P < 0.05; **, P < 0.01; and ***, P < 0.001.

**Figure:**
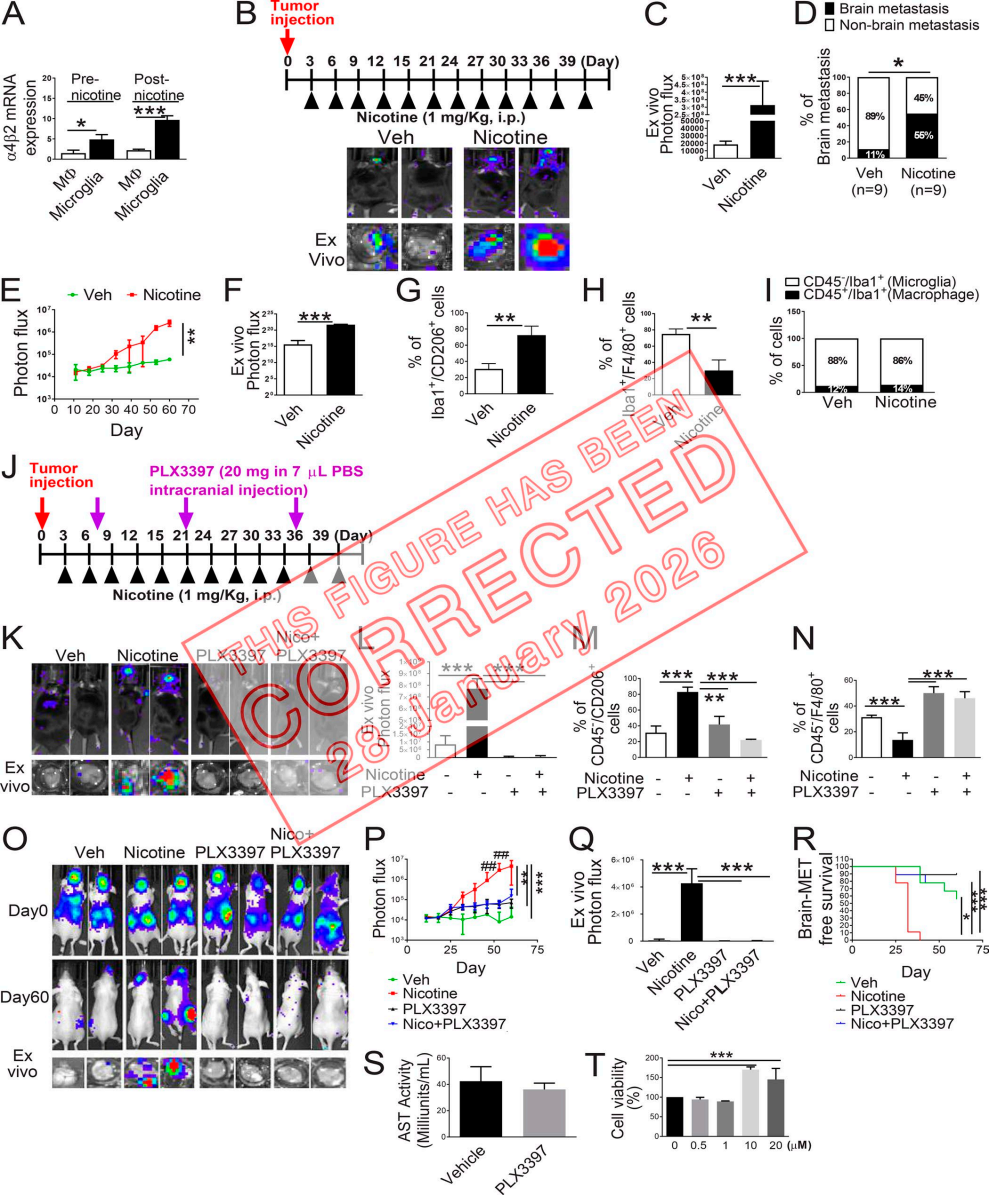


**Figure S2.**
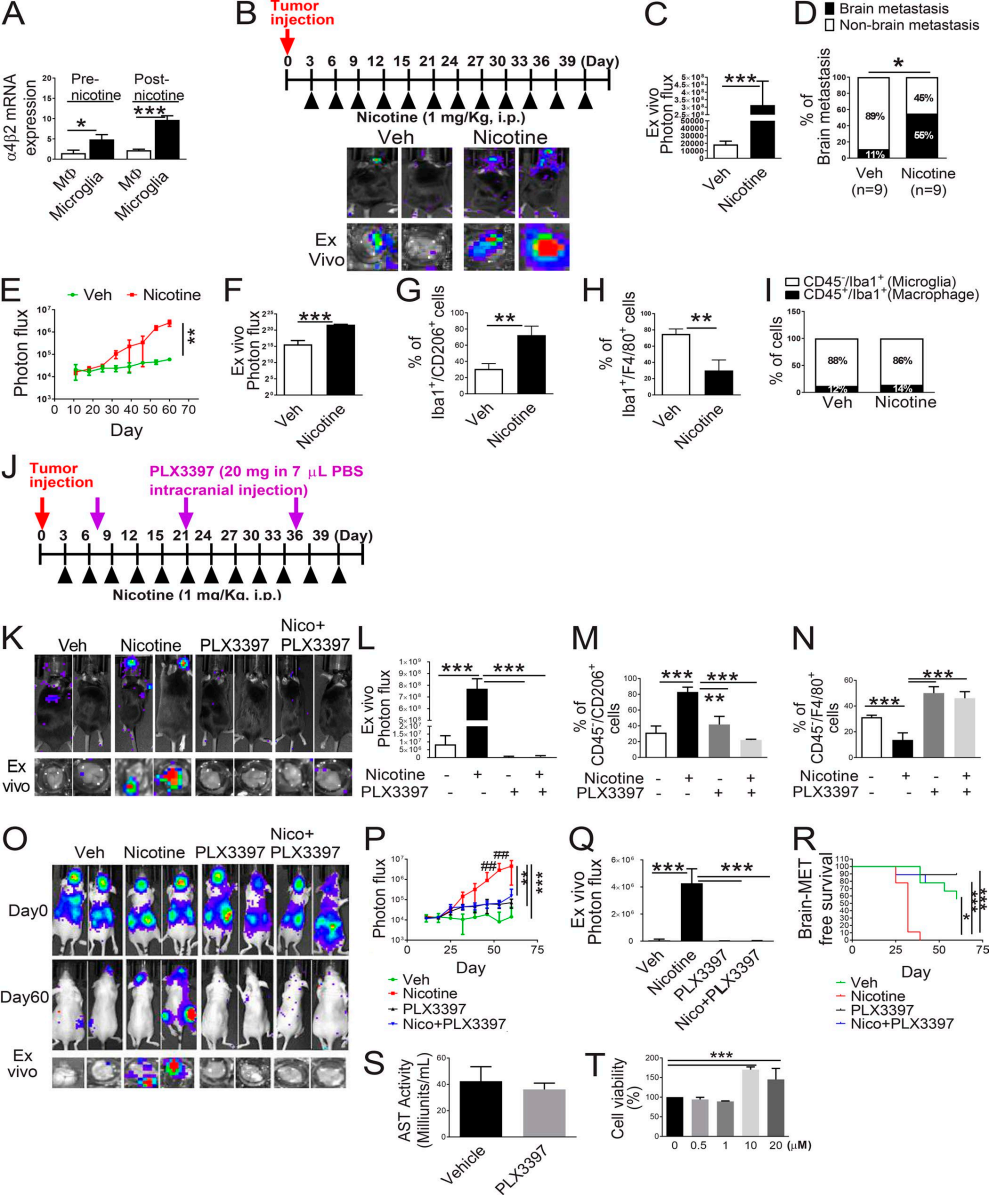
**Nicotine promotes brain metastasis in mouse model. (A)** Expression of the *α4β2* nicotine receptor on human microglia (HMC3) and human monocyte/macrophage (THP-1) were evaluated before and after nicotine treatment by qRT-PCR (*n* = 4/group). **(B)** Mouse lung cancer LL/2 cells were intracardially injected into wild-type C57BL/6 mice (*n* = 9/group). After 3 d of intracardiac transplantation of LL/2 cells, mice received nicotine (1 mg/kg) treatment by intraperitoneal injection every 3 d. Upper panels are BLI images of brain metastatic lesions of representative mice from each experimental group at day 40. The lower panels represent the total photon flux of ex vivo brain metastatic lesions as measured by BLI at the endpoint (day 40). **(C)** Quantitative data of in vivo brain metastasis of lung cancer (*n* = 9/group). **(D)** Percentage of brain metastasis of lung cancer in C57BL/6 mice with or without nicotine treatment (*n* = 9/group). **(E and F)** Quantitative data of in vivo brain metastasis of lung cancer (left panel) and ex vivo signals in the brain at the end point (right panel) of the experiment conducted in Fig. 2 B (*n* = 9/group). **(G and H)** Metastatic brain tumor tissues in Fig. 2 B were dissociated, and the effect of nicotine was examined on Iba1^+^/CD206^+^ (G) or Iba1^+^/F4/80^+^ cells (H) by FACS (*n* = 9/group). **(I)** Metastatic brain tumor tissues in Fig. 2 B were dissociated, and the Iba1^+^/CD45^−^ (microglia) and Iba1^+^/CD45^+^ (macrophage) were measured and plotted in relation to the nicotine treatment (*n* = 9/group). **(J)** Scheme of the experimental design. After 3 d of intracranial transplantation of LL/2 cells, the mice received nicotine (1 mg/kg) by intraperitoneal injection every 3 d. After 1 wk of tumor transplantation, PLX3397 (20 mg/kg in 7 µl PBS) was directly injected into the right brain of the mice every 2 wk. **(K)** Upper panels are BLI images of brain metastatic lesions of representative mice from each experimental group at day 40. The lower panels represent the total photon flux of ex vivo brain metastatic lesions as measured by BLI at the endpoint (day 40; *n* = 9/group). **(L)** Quantitative data of ex vivo brain metastasis of lung cancer at the end point. **(M and N)** Metastatic brain tumors in K were isolated from the brain and examined by FACS for M2 (M) and M1 (N) microglial polarization (*n* = 9/group). **(O)** H2030BrM (2 × 10^5^ cells) were intracardially injected into nude mice (*n* = 9/group) followed by administering them with nicotine plus PLX3397 (1 mg/kg) by intraperitoneal injection every 3 d. Upper and middle panels are BLI images of brain metastatic lesions of representative mice from each experimental group at day 0 and day 60, respectively. Lower panels represent the total photon flux of ex vivo brain metastatic lesions as measured by BLI at the endpoint (day 60; *n* = 9/group). **(P and Q)** Quantitative data of in vivo brain metastasis of lung cancer and (Q) ex vivo signals in the brain at the end point (day 60; *n* = 9/group). **(R)** Kaplan–Meier analysis of brain metastasis–free survival was performed. **(S)** Serum of mice in Fig. 2 E was collected, and an aspartate aminotransferase test (AST) was measured using the AST activity assay kit (Sigma-Aldrich) (n = 9/group). **(T)** H2030BrM cells were incubated with the indicated concentration of nicotine for 24 h. They were then examined for cell viability (*n* = 9/group). The data are presented as the mean ± SD. *, P < 0.05; **, P < 0.01; and ***, P < 0.001 versus respective nicotine group. ##, P < 0.01 versus respective PLX3397 and Nico+PLX3397 groups.

